# Primary Neuroendocrine Tumor of Urinary Bladder: A Case Report of an Unusual Occurrence

**DOI:** 10.7759/cureus.22720

**Published:** 2022-02-28

**Authors:** Bindu Rajkumar, Rishabh Sahai, Dezy Singh, Monika Singh, Ashok Singh, Arvind Kumar

**Affiliations:** 1 Department of Pathology and Laboratory Medicine, All India Institute of Medical Sciences (AIIMS) Rishikesh, Rishikesh, IND; 2 Department of Pathology and Laboratory Medicine, All India Institute of Medical Sciences (AIIMS) Gorakhpur, Gorakhpur, IND; 3 Department of Agad Tantra Evum Vidhi Vaidyaka, Uttarakhand Ayurved University (UAU) Rishikul Campus, Rishikesh, IND

**Keywords:** rare tumors, rare case report, urinary bladder tumor, primary neuroendocrine tumor, urinary bladder, primary, paraganglioma, neuroendocrine

## Abstract

Pheochromocytomas are tumors arising from catecholamine secreting cells of adrenal glands. Extra adrenal gland pheochromocytomas are called paragangliomas. They account for 15% of all pheochromocytomas. Paraganglioma arising in the urinary bladder is extremely rare accounting for 0.06% of all urinary bladder tumor cases. We present a case of a 55-year-old female patient who complained of pain in abdomen and intermittent haematuria subsequently. Magnetic resonance imaging (MRI) pelvis was done which gave the possibility of paraganglioma. An excision of bladder mass was done and sent for histopathology. On histopathology accompanied by immunohistochemistry, a final diagnosis of paraganglioma was given. The patient is on regular follow-up.

## Introduction

Chromaffin cells are derived from neural crest cells, usually located in the adrenal medulla. Tumors arising from these cells are called pheochromocytoma which is 10% having an extra-adrenal presentation [1]. Paraganglioma of the urinary bladder is a rare entity constituting <0.06% of bladder tumors. They are more common in females and usually present with hypertension, headache, palpitation, painless hematuria. Most of the paragangliomas are benign (90%) and cured completely by surgery [1,2]. Bladder paragangliomas can be asymptomatic and occasionally found during physical examination. The patient may show paroxysmal hypertension, haematuria and other clinical manifestations. Because of the low incidence and usually atypical symptoms, they are easily misdiagnosed. Surgical resection is the most effective way of treatment. However, histopathology coupled with immunohistochemistry is diagnostic for this lesion [3].

## Case presentation

**Figure 1:**
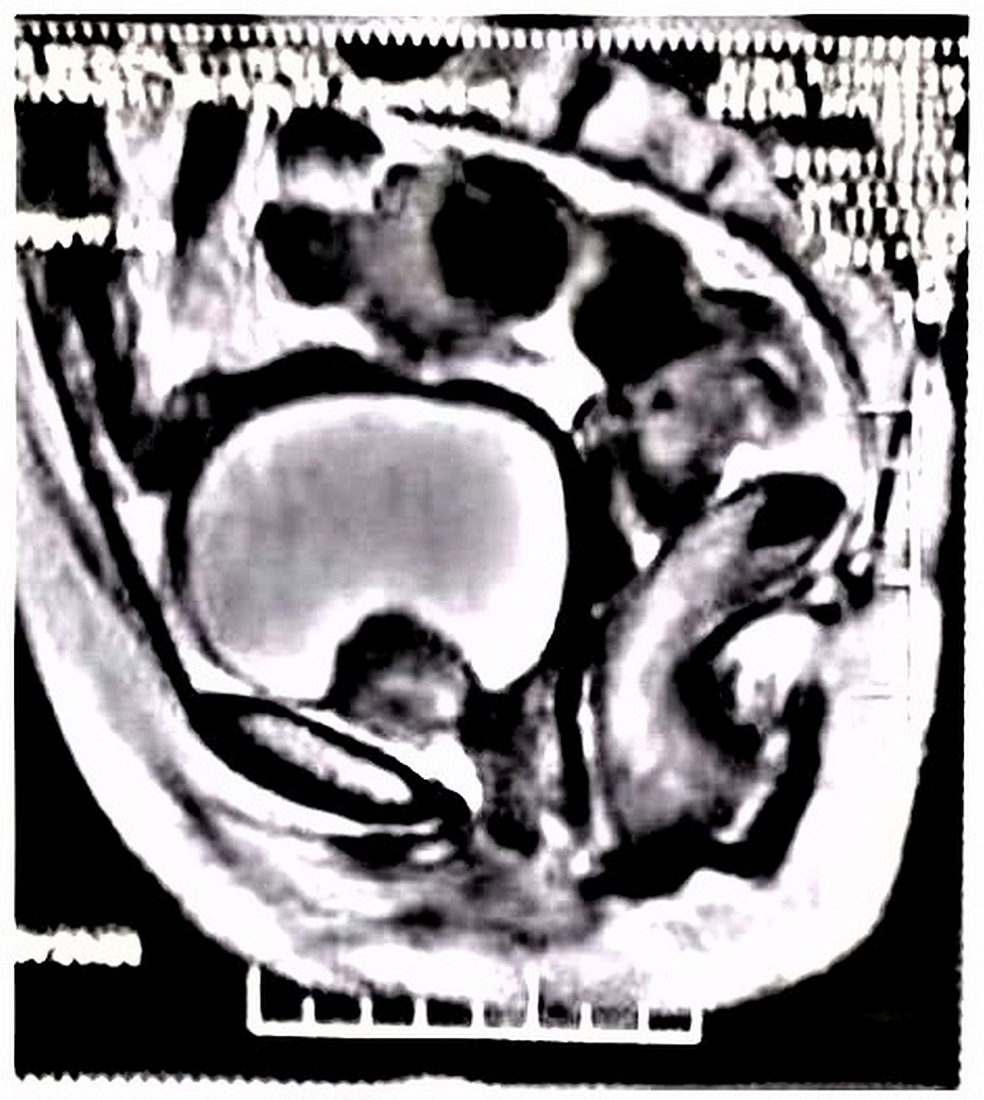
Contrast-enhanced computed tomography (CECT) image - Lobulated tumor mass arising from the base of urinary bladder and protruding into the cavity.

A 55-year-old female patient presented with diffuse abdominal pain and intermittent hematuria. Her vitals were stable. Physical and systemic examinations were unremarkable. Investigation revealed haemoglobin 12.3g/dl with normal total leucocytes count and platelets. The liver function test, kidney function test, serum electrolytes were all within normal limits. Random blood sugar was 99mg/dl. Urine routine analysis was also within normal limits. Urine for malignant cytology showed negative for any atypical cells. However, her plasma free metanephrines were elevated. Contrast-enhanced computed tomography (CECT) abdomen showed a well-defined mass measuring 41x34mm likely arising from the base of the urinary bladder and bulging into the bladder lumen (Figure 1).

**Figure 2:**
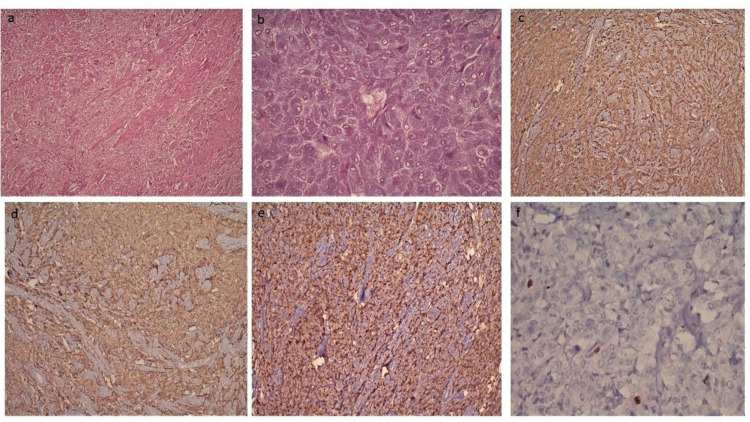
(a) Diffusely infiltrating tumor with nesting and trabecular pattern involving muscle bundles with prominent vascular network (H&E, 100X). (b) Round to oval cells with vesicular nucleus, inconspicuous nucleoli, and abundant granular eosinophilic cytoplasm along with well-defined cytoplasmic membrane (H&E, 400X). (c) Tumor cells are positive for synaptophysin (IHC, 100X). (d) Tumor cells are positive for chromogranin (IHC, 100X). (e) S-100 positivity in sustentacular cells (IHC, 100X). (f) Ki-67 labelling index <3% in tumor cell (IHC, 400X).

MRI pelvis showed a well-defined lobulated avidly enhancing submucosal bladder mass anterior to the urethral opening. Urine culture was negative. A transurethral resection of bladder tumor (TURBT) was given for histopathological examination, and the report rendered was suggestive of neuroendocrine tumor. Later on, a robotic excision of bladder mass was done and sent for histopathological examination. Grossly the specimen was yellow-tan lobulated mass measuring 3.5x3x2cm. On cutting open, homogenous greyish-yellow areas were noted. Microscopy showed tumor cells arranged in sheets and nests. Individual cells showed mild to moderate nuclear pleomorphism, with a round to oval vesicular nucleus and granular eosinophilic cytoplasm. Few of the cells show eccentrically placed nuclei. The tumor cells are seen infiltrating into the muscle bundles. No lymphovascular invasion or necrosis is seen. An occasional mitotic figure was noted (Figure 2a, 2b). Immunohistochemistry (IHC) was positive for synaptophysin, chromogranin, CD-56, with focal nuclear positivity for S-100. Proliferative marker Ki-67 was <3%. The tumor cells were negative for Pan-ck, CK-7, CK-20, HMB-45 excluding another similar morphological differential diagnosis (Figure 2c-2f).

Based on the above morphology and immunohistochemistry, a diagnosis of pheochromocytoma of the urinary bladder was made.

## Discussion

Paraganglioma is an extra-adrenal neoplasm that is derived from the chromaffin tissue of the sympathetic nervous system of the bladder wall, commonly situated at the dome or trigone of the bladder and they may be functional or non-functional. About 50% of paragangliomas are hereditary usually associated with VHL, NF1, and Carney triad [3]. Urinary bladder paragangliomas account for <1% of all paragangliomas [4]. Moreover, only 200 cases have been notified in global literature [5]. Zimmerman et al. were the first to publish a report on urinary bladder paraganglioma in the year 1953 [6]. However, in the genitourinary tract, the most common site is the urinary bladder (79.2%), followed by the urethra (12.7%), pelvis (4.9%), and ureter (3.2%) [7,8]. They commonly occur in females and are seen in the age groups between 20 to 40 years [9]. The functional paragangliomas are associated with hypertension in 1/3rd of the cases, with hematuria being the most common presentation. A study by Yamamamoto et al., which included 234 cases, showed 41.3% of patients had macroscopic hematuria, 33.2% hypertension, and 23% hypertension with seizures [10].

Priyadarshi and Pal illustrated a case of silent paraganglioma in an elderly patient who developed severe hypertension during transurethral resection [11]. On cystoscopy, the tumor appears as a submucosal yellow tumor. On histopathology, tumor cells are seen as large polygonal cells with abundant granular cytoplasm arranged in zellballen pattern and surrounded by a rich fibrous network of blood vessels [11]. Nested variants of urothelial carcinoma, granular cell tumor, malignant melanoma, metastatic renal cell carcinoma, and carcinoid tumor are the morphological differential diagnosis of bladder paraganglioma. Paragangliomas are positive for synaptophysin, chromogranin, and CD56 with low proliferation cell marker ki-67 which is <3%. A low cutoff (<10%) of Ki-67 discriminates between benign and malignant tumors. A common differential diagnosis of paraganglioma is a nested variant of urothelial carcinoma which is positive for cytokeratin and p63 immunostains. Another differential diagnosis was excluded by IHC as malignant melanoma shows positivity for HMB-45 and S100, carcinoid tumor and metastatic renal cell carcinoma by the presence of pan-cytokeratin, granular cell tumor by diffuse positivity by S100 immunostain in the tumor cell. The treatment is surgical resection by transurethral resection of bladder tumor (TURBT) or partial cystectomy. Long-term follow-up is essential.

## Conclusions

Paragangliomas of the urinary bladder are rare tumors that can mimic another urothelial neoplasm. Thus, knowledge of this entity among urologists and pathologists is necessary to avoid any misadventure. A high index of suspicion along with clinical, laboratory, and histopathology with immunohistochemistry application can differentiate this tumor from others.
